# Large-Scale Selective Sweep among *Segregation Distorter* Chromosomes in African Populations of *Drosophila melanogaster*

**DOI:** 10.1371/journal.pgen.1000463

**Published:** 2009-05-01

**Authors:** Daven C. Presgraves, Pierre R. Gérard, Anjuli Cherukuri, Terrence W. Lyttle

**Affiliations:** 1 Department of Biology, University of Rochester, Rochester, New York, United States of America; 2 Radcliffe Institute for Advanced Study, Harvard University, Cambridge, Massachusetts, United States of America; 3 Department of Cell and Molecular Biology, University of Hawaii, Honolulu, Hawaii, United States of America; Fred Hutchinson Cancer Research Center, United States of America

## Abstract

*Segregation Distorter* (*SD*) is a selfish, coadapted gene complex on chromosome 2 of *Drosophila melanogaster* that strongly distorts Mendelian transmission; heterozygous *SD/SD* ^+^ males sire almost exclusively *SD*-bearing progeny. Fifty years of genetic, molecular, and theory work have made *SD* one of the best-characterized meiotic drive systems, but surprisingly the details of its evolutionary origins and population dynamics remain unclear. Earlier analyses suggested that the *SD* system arose recently in the Mediterranean basin and then spread to a low, stable equilibrium frequency (1–5%) in most natural populations worldwide. In this report, we show, first, that *SD* chromosomes occur in populations in sub-Saharan Africa, the ancestral range of *D. melanogaster*, at a similarly low frequency (∼2%), providing evidence for the robustness of its equilibrium frequency but raising doubts about the Mediterranean-origins hypothesis. Second, our genetic analyses reveal two kinds of *SD* chromosomes in Africa: inversion-free *SD* chromosomes with little or no transmission advantage; and an African-endemic inversion-bearing *SD* chromosome, *SD-Mal*, with a perfect transmission advantage. Third, our population genetic analyses show that *SD-Mal* chromosomes swept across the African continent very recently, causing linkage disequilibrium and an absence of variability over 39% of the length of the second chromosome. Thus, despite a seemingly stable equilibrium frequency, *SD* chromosomes continue to evolve, to compete with one another, or evade suppressors in the genome.

## Author Summary

Mendel's first law of segregation holds that a heterozygous parent will transmit alternative alleles to offspring equally. *Segregation Distorter* (*SD*) is a naturally occurring selfish gene complex in *D. melanogaster* that subverts Mendel's first law. During spermatogenesis in heterozygous *SD*/*SD* ^+^ males, *SD* effectively kills *SD* ^+^-bearing sperm, monopolizing fertilization. *SD* chromosomes carry a distorter gene and a complement of genetically linked enhancers, often held together by inversions. Thus, *SD* chromosomes are selfish, co-adapted gene complexes. Although *SD* is one of our best-characterized selfish gene systems, we still have a poor understanding of its evolutionary history and population dynamics. We therefore performed a large screen for *SD* chromosomes in African populations of *D. melanogaster* and studied their genetic properties and history. We found a new *SD* chromosome type, *SD-Mal* (endemic to Africa), that has a perfect transmission advantage and lacks recombination over much of the chromosome. This new *SD* chromosome rapidly swept across sub-Saharan Africa sometime within the last ∼3,000 years. These findings show that selfish gene complexes evolve continuously to evade suppression by other genes in the genome and to compete with one another for a place in the population.

## Introduction

**Figure 1:**
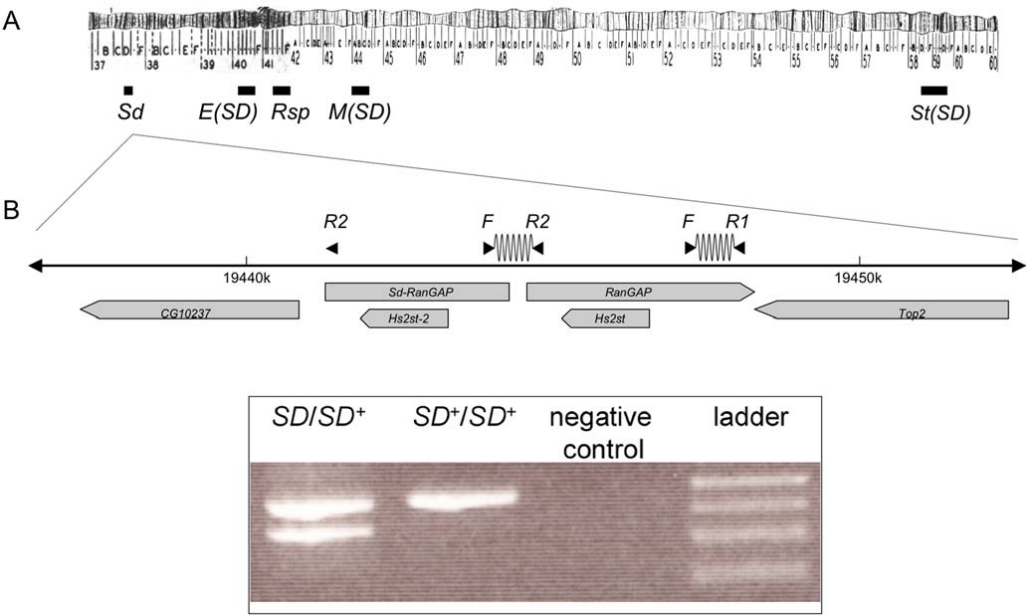
A molecular screen for *SD* chromosomes. (A) Part of chromosome arm 2L and all of 2R are shown with the approximate cytological locations of *SD* loci. The centromere occurs at the transition between cytological divisions 40 and 41. (B) A three-primer assay was used to screen isofemale lines for the presence of the *Sd-RanGAP* duplication. There are two potential primer pairs: the F-R1 primer pair, a positive control, amplifies a 463-bp product from *RanGAP*; the F-R2 primer pair amplifies a 353-bp product from the proximal breakpoint of the *Sd-RanGAP* duplicate gene, if present. Note that the R2 primer anneals to the 5′ region of both *RanGAP* and *Sd-RanGAP*; for *Sd-RanGAP*, however, there is no corresponding forward primer. An example gel is shown: flies carrying *Sd-RanGAP* yield two amplicons (from *Sd-RanGAP* and *RanGAP*), whereas those lacking *Sd-RanGAP* produce only one (from *RanGAP* only).

The *Segregation Distorter* (*SD*) system of the fruitfly, *Drosophila melanogaster*, is a naturally occurring meiotic drive complex—instead of fair Mendelian transmission, heterozygous *SD*/*SD* ^+^ males transmit *SD* chromosomes to most, if not all, progeny [1]–[8]. Full strength distortion is caused by three interacting loci clustered around the centromere of chromosome 2 (an autosome): the *trans*-acting *Segregation distorter* (*Sd*) locus; an upward modifier, *Enhancer of SD* (*E(SD)*); and a *cis*-acting distortion-*insensitive* allele at the target locus, *Responder* (*Rsp* ^i^). (By convention, *Sd* refers to the locus whereas *SD* refers to chromosomes assumed to carry the full complex of loci.) *SD* chromosomes are thus *Sd E(SD) Rsp* ^i^, whereas *SD* ^+^ chromosomes, which lack the distorting *Sd* locus and usually carry *sensitive* alleles of *Rsp*, are *Sd* ^+^ *E(SD)* ^+^ *Rsp* ^s^ (Figure 1A). During spermiogenesis in heterozygous *SD*/*SD* ^+^ males, the sperm-specific histone transition required for proper chromatin packaging is disrupted in *Rsp* ^s^-bearing *SD* ^+^ sperm, leaving functional *Rsp* ^i^-bearing *SD* sperm to monopolize fertilization [9]–[12]. For decades, the *SD* system has been a model in evolutionary genetics, not only for being *selfish*, propagating at the expense of its bearers, but as a *coadapted gene complex* whose fitness is determined by multiple epistatic interactors [5]–[7], [13]–[15].

The evolution and persistence of the *SD* complex depend critically on genetic linkage. Multilocus drive systems can only invade a population when recombination is restricted among loci, as the transmission advantage of distorter chromosomes (*Sd Rsp* ^i^) must not be offset by the formation of so-called ‘suicide’ chromosomes (*Sd Rsp* ^s^) that distort against themselves [16]. The clustering of *SD* loci around the centromere of chromosome 2, where crossing over is reduced, is therefore unsurprising [15]. Epistatic selection further favors the evolution of secondary suppressors of recombination [15],[17],[18]. Many *SD* chromosomes, for instance, have recruited a pericentric inversion, *In(2LR)39D-42A*, that further reduces crossing over in the centromeric region, while some have recruited paracentric inversions on 2R (reviewed in [2],[5],[6],[17]). The paracentric inversions are thought to reduce crossing over between the centromeric *SD* elements and modifiers of distortion distributed across 2R, such as *Modifier of SD* (*M(SD)*), *Stabilizer of SD* (*St(SD)*), and possibly others [19]–[22]. Thus, *SD* chromosomes have evolved a complex of multiple, epistatically interacting loci with coadapted alleles whose linkage relationships are usually further tightened by one or more chromosomal inversions.

The geographic distribution of inversions on different *SD* chromosomes may shed light on the origins, and possibly the age, of the complex. *SD* can be found in nearly all populations of *D. melanogaster* at a frequency of ∼1–5% [23] (but see ref. [24]). In North America, Hawaii, Japan, and Australia, *SD* chromosomes invariably carry inversions (though not necessarily the same ones). In Italy and Spain, however, both inversion-bearing and presumably ancestral, inversion-free *SD* chromosomes occur. The presence of both derived and ancestral types has been taken as evidence that *SD* chromosomes originated in the Mediterranean basin [3],[4]. An origin in Mediterranean Europe further implies that the *SD* complex evolved recently, as *D. melanogaster* is a sub-Saharan African species whose range expanded to Europe only ∼15,000 years ago, probably via a single major out-of-Africa founder event [25]–[28]. The first population genetic analysis of *SD* found little divergence between four loci on *SD* versus *SD* ^+^ chromosomes, consistent with a recent origin for the complex [14],[29].

Much about the evolutionary history and population dynamics of *SD* in natural populations remains unclear. For one, a recent, Mediterranean origin for *SD* in the *D. melanogaster* lineage has important implications, explaining its absence from closely related species and suggesting that the multiple genetic components of the complex evolved very quickly. But the Mediterranean origins hypothesis hinges on few data—the presence of inversion-free *SD* chromosomes from collections in Italy and Spain and nowhere else. For another, what little is known about the population dynamics of *SD* comes from laborious, large-scale phenotypic assays to determine the frequency of *Sd* and *Rsp* in natural populations (e.g., [30],[31]). These have revealed that in natural populations of *D. melanogaster* worldwide, the frequency of *SD* is remarkably similar (1–5%) and thus presumably stable. The stability of *SD* occurs because its intrinsic transmission advantage is balanced by several forces: the sterility of many *SD/SD* males [32]; the reduced sperm numbers in *SD*-bearing flies [33]; the presence of suppressors of distortion [24],[30],[31],[34]; and selection against linked deleterious mutations that accumulate in the large non-recombining regions of *SD* chromosomes [1]. This apparent stability may however mask an underlying evolutionary turnover among competing *SD* chromosomes predicted by theory [15]. Using realistic parameters, Charlesworth and Hartl [15] showed that an inversion-free *SD* chromosome will invade a *SD* ^+^ population and spread to a low-frequency equilibrium; this mixed population, however, is susceptible to invasion by an inversion-bearing *SD* chromosome that will displace the inversion-free *SD* and spread to the same low-frequency equilibrium. It appears, then, that *SD* chromosomes may evolve continuously, as a small subpopulation of second chromosomes in *D. melanogaster*, competing with one another and evading suppressors.

Fifty years of *SD* work has produced a rich body of genetics and theory [2], [5]–[8], and recently the molecular basis of distortion has begun to emerge [8], [35]–[38]. The two main *SD* loci have been identified: *Sd* is a partial, tandem duplication of the gene *RanGAP*, called *Sd-RanGAP* [37]; and *Rsp* is a large array of 120-bp AT-rich repeats in the centric heterochromatin of 2R [38], where alleles with ≤300 repeats are “insensitive” (*Rsp* ^i^), 700–1100 are “sensitive” (*Rsp* ^s^), and ≥1100 are “super-sensitive” (*Rsp* ^ss^; [3],[4],[38]). *Sd-RanGAP* encodes truncated RanGAP (Ran-GTPase Activating Protein; [37]), a protein with essential and evolutionarily conserved functions in nuclear transport, mitosis, and chromatin processing [39]–[41]. The truncated Sd-RanGAP protein is enzymatically active but mislocalizes to the nucleus (normally RanGAP is cytoplasmic) where, for reasons not yet clear, it causes segregation distortion in *SD*/*SD* ^+^ males [8],[35],[36].

Surprisingly, in the decade since its discovery [37], there have been no direct evolutionary analyses of *Sd-RanGAP*, the gene that actually causes distortion. In this paper, we study the molecular population genetics of the *SD* complex to investigate its evolutionary history and recent population dynamics. First, we perform the first screen for *SD* in populations from Africa, the ancestral range of *D. melanogaster*. Second, we study patterns of DNA sequence variation at the distorter, *Sd-RanGAP*, as well as its parent gene, *RanGAP*, and eight noncoding loci on chromosome 2. Finally, we characterize the strength of distortion, inversion status, and mutational load of *SD* chromosomes. We show that *Sd-RanGAP* is present in Africa and that a new *SD* chromosome type has spread very recently across the African continent, causing a large-scale selective sweep among *SD* chromosomes. These results call into question our current understanding of the timing and location of *SD*'s origins and suggest that, despite its remarkably stable population frequency, *SD* evolution is not at equilibrium.

## Results

### A Screen for *SD* Chromosomes in African Populations

We used a three-primer PCR assay to screen 452 isofemale lines collected from 13 localities in Africa for the *Sd-RanGAP* duplication (Figure 1B; Table 1). We found 12 *SD* chromosomes from across the continent, including west (*e.g.*, Benin, Gabon, Cameroon) and east Africa (*e.g.*, Zimbabwe, Kenya; Table 2). Assuming that all isofemale lines are homozygous, the population frequency of *SD* is 12/452 = 0.027; and assuming that all isofemale lines are heterozygous, the population frequency of *SD* is 12/904 = 0.013. These estimates suggest that *SD* chromosomes occur in Africa at a frequency of 1.3–2.7%, similar to its frequency in other natural populations [23].

**Table 1:** Isofemale lines screened for *SD* chromosomes.

Geographic origin	Lines screened	*SD* chromosomes
Benin	7	1
Cameroon	132	3
Congo	15	0
Eritrea	26	0
Gabon	32	1
Ghana	2	0
Kenya	41	5
Malawi	19	0
Niger	44	0
Nigeria	10	0
South Africa	13	0
Uganda	37	0
Zimbabwe	74	2
Total	452	12

**Table 2:** *SD* chromosomes from Africa.

*SD* chromosome	Geographic origin	Inversions^a^
*SD-GN09*	Gabon	*In(2R)Mal*
*SD-KM87*	Malindi, Kenya	*In(2L)t+In(2R)Mal*
*SD-KM92*	Malindi, Kenya	*In(2R)Mal*
*SD-KN20*	Kenya	*In(2R)Mal*
*SD-KY38*	Malindi, Kenya	*In(2L)t+In(2R)Mal*
*SD-KY91*	Kenya	*In(2R)Mal*
*SD-MD21*	Mbalang-Djalingo, Cameroon	*In(2R)Mal*
*SD-NK04*	Nkouondja, Cameroon	*In(2R)Mal*
*SD-ZK178*	Lake Kariba, Zimbabwe	*In(2L)t+In(2R)Mal*
*SD-ZK216*	Lake Kariba, Zimbabwe	*In(2L)t+In(2R)Mal*
*SD-BN19*	Benin	No Inversions
*SD-MD31*	Mbalang-Djalingo, Cameroon	No Inversions

a *In(2R)Mal* has cytological breakpoints 44F3–12;54E3–10+51B6–11;55E3–12, and *In(2L)t* has breakpoints 22D3–22E1;34A8–34A9.

### Low Divergence between *Sd-RanGAP* and *RanGAP*

**Figure 2:**
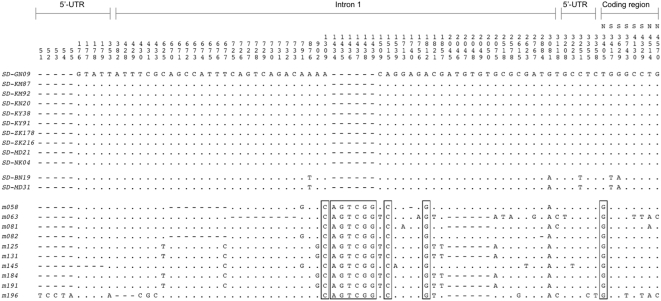
Variation in 12 *Sd-RanGAP* and 10 *RanGAP* sequences from African populations of *D. melanogaster*. *Sd-RanGAP* and *RanGAP* show four nucleotide differences and one indel difference. (N = nonsynonymous; S = synonymous; all other changes are noncoding.)

After genetically extracting the *SD* chromosomes, we sequenced the ∼4.5-kb *Sd-RanGAP* sequence from all 12 as well as the homologous region of the parent gene, *RanGAP*, from 10 wildtype (non-*SD*) chromosomes sampled from Zimbabwe (see Methods). *RanGAP* and *Sd-RanGAP* show typical levels of silent divergence per site from the *RanGAP* homolog in the outgroup species, *D. simulans*, with *K*_sil_ = 0.0471 and 0.0478, respectively. Silent divergence between the duplicate genes, *RanGAP* and *Sd-RanGAP*, within *D. melanogaster* is more than an order of magnitude lower, *K*_sil_ = 0.0027 (see also ref. [37]). These findings confirm that *Sd-RanGAP* arose in *D. melanogaster* well after the split from *D. simulans* [29]. Using *D. simulans RanGAP* as an outgroup sequence, we polarized the substitutions between *D. melanogaster RanGAP* and *Sd-RanGAP*. Of five fixed differences between *RanGAP* and *Sd-RanGAP*, all were fixed in the common ancestor of the *Sd-RanGAP* sequences: three noncoding changes, one fixed 6-bp deletion, and a single nonsynonymous change (Figure 2). The first intron of *RanGAP* contains the gene, *Hs2st*, raising the possibility that some “silent” changes in one gene are not silent in the other. However, of the five fixed substitutions occurring in *Sd-RanGAP*, only four affect *Hs2st*: two are noncoding and two are synonymous.

### DNA Sequence Variation at *RanGAP* and *Sd-RanGAP*

The amount and distribution of DNA sequence variability at *RanGAP* is not unusual for an autosomal locus sampled from African populations of *D. melanogaster*. First, among the 10 wildtype *RanGAP* sequences, we detect 29 segregating sites (Figure 2), with two measures of DNA sequence polymorphism per site, *π* = 0.0020 and *θ* = 0.0023. These values show that *RanGAP* harbors less variability than the average autosomal locus in African populations (*π* = 0.0104 and *θ* = 0.0114; ref. [27]), but this is not unexpected as *RanGAP* resides in a centromere-proximal region (37E) with a relatively low rate of crossing over and is thus especially susceptible to background selection and hitchhiking effects [42],[43]. Three polymorphisms are synonymous, two are nonsynonymous, and 23 are noncoding, with 66% falling in the large first intron (3.2 kb). The site frequency spectrum at *RanGAP* does not deviate significantly from standard neutral expectations (Tajima's *D* = −0.606, *P* = 0.297; Fay and Wu's *H* = −4.711, *P* = 0.127 [44],[45]), where significance was evaluated from 10,000 coalescent simulations conditioning on the observed *θ* and assuming no recombination). The moderately negative Tajima's *D* is consistent with recent expansion in the African *D. melanogaster* populations as inferred from other autosomal loci [27].

*Sd-RanGAP* is less variable than *RanGAP*: among the 12 *Sd-RanGAP* sequences, we detect only five segregating sites (Figure 2), with *π* = 0.0003 and *θ* = 0.0004, and a site frequency spectrum skewed towards a moderate excess of rare variants, although not significantly (Tajima's *D* = −0.313, *P* = 0.395; Fay and Wu's *H* = −0.242, *P* = 0.250). Of the five polymorphisms, two are synonymous and three are noncoding. The lower variability at *Sd-RanGAP* relative to *RanGAP* is, of course, expected as *Sd-RanGAP* is present on only ∼2% of second chromosomes. There are no shared polymorphisms between *RanGAP* and *Sd-RanGAP* and hence no evidence for recent gene conversion due to ectopic recombination [46]. The lack of recombination between the two loci implies that *Sd-RanGAP* evolves as an isolated subpopulation of sequences with a distinct genealogical history.

### Unusual Haplotype Structure at *Sd-RanGAP*

We found six haplotypes among the ten wildtype *RanGAP* sequences, with levels of linkage disequilibrium (LD) typical of an autosomal locus in Africa (*Z_nS_* = 0.247; [27],[47]). In contrast, the spatial distribution of polymorphic sites in the 4.5-kb *Sd-RanGAP* sequences is unusual: mutations at five segregating sites are in perfect linkage disequilibrium (*Z_nS_* = 1.0), forming just two haplotypes (*K* = 2). The major haplotype occurs ten times in the sample (*M* = 10) and the minor haplotype twice. We used coalescent-based haplotype configuration tests to estimate the probability of observing such unusual haplotype structure under standard neutral model assumptions [48]. We performed 100,000 coalescent simulations without recombination (a conservative assumption), assuming a sample size of *n* = 12 and five segregating sites (*S* = 5). The cumulative probability that the observed haplotype configuration, or one more extreme, occurs by chance is *P* = 0.0378. Two features of the haplotype configuration, in particular, differ significantly from the expectations of a neutral genealogical process: the major haplotype is too common in the sample, *P*(*M*≥10|*n* = 12, *S* = 5) = 0.0313; and there are too few kinds of haplotypes, *P*(*K*≤2|*n* = 12, *S* = 5) = 0.0285. Both features of the data are consistent with an incomplete selective sweep in which the major haplotype has quickly and recently risen to high frequency, but not fixation, among *SD* chromosomes [49].

### Large-Scale Selective Sweep among African *SD* Chromosomes

**Figure 3:**
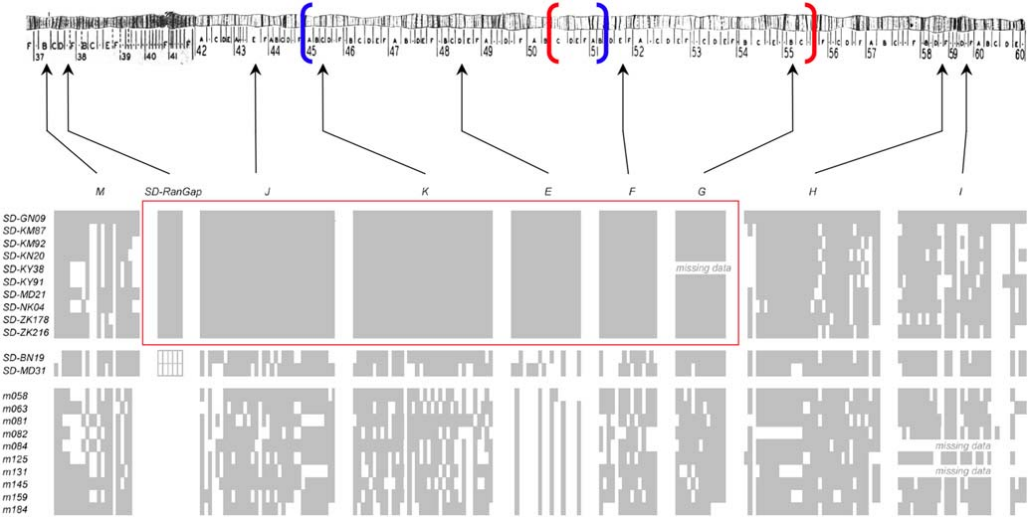
Distribution of DNA sequence variation at *Sd-RanGAP* and eight non-coding regions in Africa. Sequences were sampled from 12 *SD* and 10 wildtype second chromosomes from African populations of *D. melanogaster*. The positions of the two overlapping inversions, *In(2R)Mal*, and the sequenced loci are shown on chromosome 2 (only part of 2L is shown). Sequence variants are arbitrarily coded: gray matches *SD-GN09*, white does not. The red box highlights the long, mutation-free haplotype that spans from *Sd-RanGAP* on 2L to locus *G* (55B) on 2R.

If the major African *SD* haplotype has indeed risen to high frequency due to positive selection or superior segregation distortion, then the haplotype structure may extend beyond the *Sd-RanGAP* region. To test this possibility, we sequenced eight noncoding regions across chromosome 2 from all 12 African *SD* chromosomes and from 10 wildtype chromosomes (Figure 3; Table 3). The amount and distribution of DNA sequence variation among wildtype chromosomes was typical for African *D. melanogaster* populations [27], with *θ* ranging from 0.0053 to 0.0137 and Tajima's *D* ranging from −1.630 to 0.445 (*P* = 0.045 for locus *G*, but ≥0.05 for other loci; Table 3). In addition, there is ample evidence for recombination in all but one of the loci (region *E* has an unusual lack of variability and a small number of haplotypes, though not significantly so; Table 3; Figure 3).

**Table 3:** Summary of molecular population genetic analyses at Sd-RanGAP and eight non-coding regions on chromosome 2.

	locus	*M*	*SD*	*J*	*K*	*E*	*F*	*G*	*H*	*I*
	gene	*tup*	*Sd-RanGAP*	*CG30497*	*Myd88*	*off-track*	*scab*	*staufen*	*plexus*	*CG34372*
	region	p_IGR	gene	intron	d_IGR	p_IGR	p_IGR	p_IGR	intron	d_IGR
	cytol. location	37B	37E	43E	45C	48D6	51E	55B	58E	59E
Wildtype (non-*SD*)	n	10	-	10	10	10	10	10	10	8
	L	707	-	834	824	715	612	567	728	626
	*S*	14 (15)	-	18	31 (32)	13	12	12	11	20
	*π*	0.0092	-	0.0062	0.0124	0.0073	0.0076	0.0048	0.0042	0.0110
	*θ*	0.0075	-	0.0076	0.0137	0.0066	0.0069	0.0075	0.0053	0.0123
	Tajima's *D*	1.050	-	−0.853	−0.483	0.445	0.405	−1.630 *	−0.997	−0.554
	Fay & Wu's *H*	−1.067	-	1.156	4.356	0.800	−0.978	−3.022	−1.689	−0.786
	*Z_nS_*	0.299	-	0.109	0.143	0.280	0.269	0.142	0.096	0.147
	*K*_s_	0.042	-	0.063	0.085	0.056	0.020	0.039	0.024	0.034
*SD*	*n*	12	12	12	12	12	12	11 **	12	12
	L	708	4515	865	825	700	612	567	734	626
	*S*	17	5	11	10	12	7	0	13 (14)	24
	*π*	0.0075	0.0003	0.0024	0.0024	0.0042	0.0021	0.0000	0.0051	0.0114
	*θ*	0.0080	0.0004	0.0042	0.0040	0.0057	0.0038	0.0000	0.0063	0.0127
	Tajima's *D*	−0.263	−0.313	−1.755 *	−1.705 *	−1.077	−1.713 *	-	−0.794	−0.462
	Fay & Wu's *H*	−0.818	−0.242	−4.697 *	−7.424 *	−7.242 *	−4.091 *	-	0.061	0.485
	*Z_nS_*	0.147	1.000	0.497	0.652	0.651	0.466	-	0.088	0.162
	*K*_s_	0.042	0.048	0.064	0.084	0.057	0.018	0.037	0.025	0.034
*SD-Mal* only	n	10	10	10	10	10	10	9 **	10	10
	L	708	4515	874	825	709	614	573	737	626
	*S*	14	0	0	0	0	0	0	13 (14)	23
	*π*	0.0071	0	0	0	0	0	0	0.0058	0.0121
	*θ*	0.0070	0	0	0	0	0	0	0.0067	0.0130
	Tajima's *D*	0.047	-	-	-	-	-	-	−0.631	−0.335
	Fay & Wu's *H*	−1.244	-	-	-	-	-	-	0.267	0.711
	*Z_nS_*	0.199	-	-	-	-	-	-	0.099	0.184
	*K*_s_	0.042	0.048	0.064	0.085	0.057	0.019	0.037	0.025	0.034

*: *P*<0.05, determined by 10,000 coalescent simulations conditional on observed *θ* and assuming no recombination.

**: The *SD-KY38* chromosome has a lethal mutation in the *staufen* region (G) and thus could not be sequenced from *Df(2R)Pcl7B/SD-KY38* or *Df(2R)Pcl11B/SD-KY38* flies.

p_IGR and d_IGR = proximal and distal intergenic region, respectively.

n = number of chromosomes sampled.

L = gapless length of sequenced region.

*S* = number of segregating sites (mutations) in the sample.

*π* = average number of pairwise differences per site.

*θ* = Watterson's measure of variability based on the number of segregating sites.

*Z*_nS_ = average linkage disequilibrium among all pairwise combinations of *S* segregating sites in sequenced region.

*K*_s_ = Jukes-Cantor corrected silent divergence per silent site between sample and *D. simulans*.

The distribution of variation among *SD* chromosomes differs strikingly from wildtype chromosomes in two ways. First, the frequency spectra at several loci show patterns consistent with a recent selective sweep. Of the eight noncoding regions surveyed, three loci (*J*, *K*, and *F*) show significant excesses of rare variants (Tajima's *D*; Table 3), four contiguous loci (*J*, *K*, *E* and *F*) show significant excesses of high-frequency derived variants (Fay and Wu's *H*; Table 3), and a fifth contiguous locus (*G*) possesses no variability at all. The three loci whose frequency spectra do not deviate from neutral expectations include the most distal locus on 2L (*M* at 37B) and the two most distal loci on 2R (*H* and *I* at 58E and 59E, respectively). Second, and more striking, the haplotype structure seen at *Sd-RanGAP* extends across most of chromosome arm 2R: the 10 major *Sd-RanGAP* chromosomes possess a single identical haplotype that extends from cytological region 37E on 2L (*Sd-RanGAP*) to region 55B on 2R (locus *G*; Table 3; Figure 3). The long distance LD does not extend to the most distal locus on 2L (locus *M*) or the two most distal loci on 2R (*H* and *I*; Table 3; Figure 3). Among all 12 *SD* chromosomes, forty-five segregating sites occur at *Sd-RanGAP* and the six regions extending to cytological subdivision 55B. Remarkably, all are differences between the major and minor *SD* chromosomes (17) or between the two minor *SD* chromosomes (28). The 10 major *SD* haplotypes are identical—there is not a single polymorphism in >8.1 kb of sequence. A haplotype configuration test assuming *n* = 12, *S* = 45, and no recombination confirms that this haplotype configuration (*M* = 10, *K* = 3) is highly unusual under a standard neutral genealogical process (*P* = 0.00002): the major haplotype is too common in the sample (*P*[*M*≥10|*n* = 12, *S* = 45]≤0.00001) and there are too few kinds of haplotypes (*P*[*K*≤3|*n* = 12, *S* = 34] = 0.00002). The chromosomal region between *Sd-RanGAP* and region 55B (*G*) spans ≥14 Mb and ∼30 cM, comprising more than 39% of the euchromatic length of chromosome 2. Taken together, the significantly skewed frequency spectra and the existence of an extraordinarily long, high-frequency, mutation-free haplotype suggest a large-scale selective sweep in progress among *SD* chromosomes [49].

### The Major *SD* Haplotype Recombines Less and Distorts More

**Figure 4:**
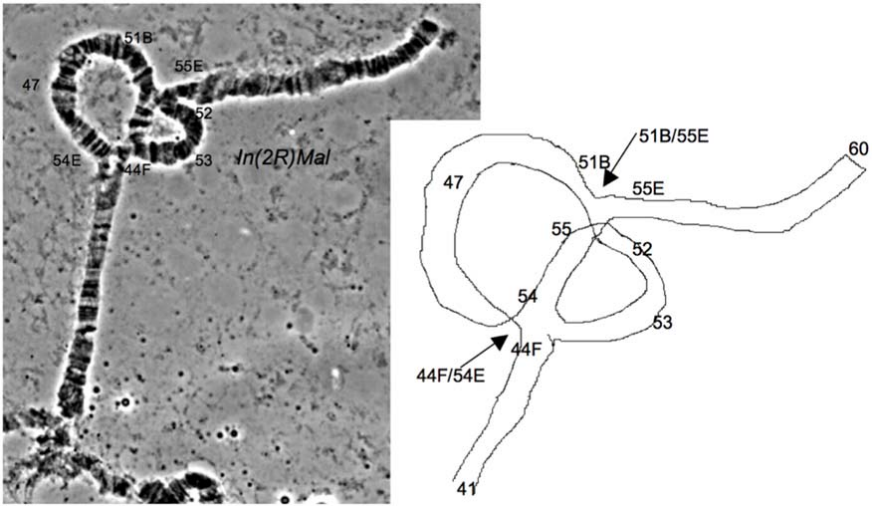
Polytene chromosome squashes reveal two overlapping inversions on arm 2R of ten *SD* chromosomes. The *In(2R)Mal* arrangement is endemic in African populations and involves two inversions, *In(2R)51B6–11;55E3–12* and *In(2R)44F3–12;54E3–10*.

The magnitude of a selective sweep is determined by two key parameters: the local rate of recombination and the strength of selection driving the major haplotype to high frequency. The *SD* chromosomes carrying the major haplotype are unique in both respects. First, recombination is suppressed along much of the major *SD* haplotype. We cytogenetically characterized the 12 African *SD* chromosomes by crossing *SD*/*SD* or *SD*/*CyO* males to virgin *cn bw* females (which are homozygous for standard-arrangement second chromosomes) and examined polytene chromosome squashes from larval salivary glands. None of the African *SD* chromosomes possess the *In(2R)NS* inversion (52A2–52B1;56F9–56F13) found on most non-African *SD* chromosomes [2]. Indeed, *SD*-*BN19* and *SD*-*MD31* are inversion-free chromosomes (Table 2). The other ten *SD* chromosomes, however, possess a complex chromosomal arrangement on 2R (Table 2). The cytological order (40–44F|54E–55E|51BC–44F|54E–51BC|55E–60) shows that these *SD* chromosomes have recruited two overlapping inversions: *In(2R)51BC;55E* first, followed by *In(2R)44F;54E* (Figure 4). These inversion breakpoints match a previously identified, but rare, African endemic chromosomal arrangement found in Malawi [50], hereafter called *In(2R)Mal*. In addition to *In(2R)Mal*, four *SD* chromosomes (*SD-KN20*, *SD-KY87*, *SD-ZK178, SD-ZK216*) carry the cosmopolitan *In(2L)t* inversion (22D3–D6;34A8–A9; Table 2).

The association between major haplotype and the *In(2R)Mal* arrangement is perfect: all major haplotype *SD* chromosomes carry *In(2R)Mal*, whereas both minor haplotype *SD* chromosomes (*SD*-*BN19* and *SD*-*MD31*) lack *In(2R)Mal* (Fisher's Exact *P* = 0.015). Hereafter, we refer to this new class of *In(2R)Mal*-bearing, major haplotype *SD* chromosomes as *SD-Mal*. To test the effect of the *In(2R)Mal* arrangement on crossing over, we crossed heterozygous *SD-NK04*/*cn bw* females (*SD-NK04* is a *SD-Mal* chromosome) to *cn bw* males and recorded the frequency of recombination between *cn* (43E16) and *bw* (59E2). As negative controls, we crossed heterozygous *SD-MD31*/*cn bw* females to *cn bw* males (*SD-MD31* is inversion free). Among progeny from the *SD-MD31* control crosses with a standard arrangement second chromosome, 34.8% carried recombinant chromosomes (*n* = 2,155 progeny). In contrast, the *In(2R)Mal* arrangement almost entirely eliminates crossing over between *cn* and *bw*: among progeny from crosses with the *In(2R)Mal*-bearing *SD-NK04* chromosome, only 0.2% carried recombinant chromosomes (*n* = 1,564). By restricting recombination with wildtype chromosomes, *In(2R)Mal* sequesters a large piece of chromosome arm 2R as an effectively non-recombining region. The lack of recombination helps to explain the long-range LD produced by the selective sweep (Figure 3) as well as the strong population differentiation at loci between *SD-Mal* and inversion free chromosomes (*S*_nn_ = 1.0, *P*≤0.0001 [51], for the *Sd-RanGAP* to 55B regions concatenated; Table 4).

**Table 4:** Genetic differentiation between *SD* and non-*SD* chromosomes.

Population comparison		*M*	*J*	*K*	*E*	*F*	*G*	*H*	*I*
		37B	43E	45C	48D6	51E	55B	58E	59E
*SD* (12) vs. wildtype (10)	*S*_nn_	0.655	0.841**	0.864*	0.848**	0.777**	0.541*	0.314	0.462
	*F*_st_	0.119	0.406	0.423	0.599	0.088	0.032	−0.024	−0.019
*SD-Mal* (10) vs. wildtype (10)	*S*_nn_	0.620	1.000**	0.950**	1.000**	0.855**	0.514	0.379	0.373
	*F*_st_	0.081	0.581	0.529	0.771	0.230	0.032	−0.012	−0.043

*P*<0.001 and ** *P*<0.0001, where probabilities determined by 10,000 permutations of the data.

We next assayed the strength of segregation distortion by estimating *k*, the proportion of progeny inheriting *SD* chromosomes from heterozygous *SD*/*Rsp* ^s^ males. In preliminary work, we found that the dominantly marked balancer chromosome, *In(2LR)Gla* (hereafter, *Gla*), carries a sensitive *Responder* (*Rsp* ^s^). We therefore measured transmission from heterozygous *SD*/*Gla* flies (see Methods). Surprisingly, the two *SD* chromosomes bearing the minor haplotype showed no detectable distortion: k* = 0.538±0.025 for *SD-BN19* and k* = 0.415±0.012 for *SD-MD31* (mean k*±s.e. are corrected for viability; Table 5). *SD-BN19* and *SD-MD31* chromosomes also failed to cause distortion when heterozygous against the super-sensitive *Rsp* ^ss^ allele of the *lt pk cn bw* chromosome (not shown). In contrast, males heterozygous for *SD-Mal* chromosomes collectively sired 10,664 progeny and failed to produce a single *Rsp* ^s^-bearing offspring (k* = 1.0; Table 5). The genetic and phenotypic data on recombination and distortion thus provide a clear explanation for the rise of the major haplotype-bearing *SD-Mal* chromosomes in Africa: they recombine less and distort more.

**Table 5:** Strength of segregation distortion for African *SD* chromosomes.

Chromosome	Male transmission					Female transmission		
	*k*_m_	±s.e.	*k*_m_*	±s.e.	Total progeny	*k*_f_	±s.e.	Total progeny
*SD-GN09*	1.000	0.000	1.000	0.000	682	0.539	0.021	487
*SD-KM87*	1.000	0.000	1.000	0.000	1124	0.561	0.037	431
*SD-KM92*	1.000	0.000	1.000	0.000	1092	0.616	0.087	799
*SD-KN20*	1.000	0.000	1.000	0.000	1072	0.595	0.024	1020
*SD-KY38*	1.000	0.000	1.000	0.000	837	0.519	0.046	664
*SD-KY91*	1.000	0.000	1.000	0.000	1544	0.579	0.064	230
*SD-MD21*	1.000	0.000	1.000	0.000	997	0.486	0.05	752
*SD-NK04*	1.000	0.000	1.000	0.000	1167	0.525	0.037	457
*SD-ZK178*	1.000	0.000	1.000	0.000	842	0.533	0.023	967
*SD-ZK216*	1.000	0.000	1.000	0.000	1307	0.555	0.034	813
*SD-BN19*	0.569	0.025	0.538	0.025	1179	0.613	0.074	561
*SD-MD31*	0.531	0.012	0.415	0.012	1531	0.621	0.035	1167
*cn bw*	0.602	0.030	0.504	0.033	580	0.615	0.036	912
*OreR*	0.654	0.024	0.558	0.026	912	0.615	0.013	876

*k*_m_ and *k*_f_ = proportion of progeny inheriting *SD* when transmitted by males and females, respectively.

*k*_m_* = viability-corrected estimate of proportion of progeny inheriting *SD* transmitted by males.

### The Age of the *SD* Sweep

The complete absence of even low frequency polymorphisms in ∼8.1 kb of sequence distributed from *Sd-RanGAP* on 2L to cytological subdivision 55B on 2R (*G*) suggests that *SD-Mal* rose to high frequency among *SD* chromosomes quickly and recently. To obtain estimates of the upper 95% confidence limit for the age of the sweep, we assumed that the genealogy relating *SD-Mal* haplotypes is star-shaped, as expected for a selective sweep, and then estimated the time back to their most recent common ancestor [52]. The expected number of segregating sites in such a sample is *E*(*S*) = *ntu*, where *n* = number of lineages, *t* = time in the past when the lineages coalesce into a single common ancestor, and *u* = the total mutation rate of the sequenced regions. Assuming that the number of mutations on the ten lineages is Poisson distributed, we numerically solved for the probability of observing zero polymorphisms, *P*(*S* = 0) = e^−*ntu*^, for different times to the common ancestor, *t*. We used two different estimates of the sequence-specific mutation rate. First, we estimated the mutation rate per generation from *θ*, which equals 4*N*_e_ *u* under standard neutral assumptions, estimated from the wildtype sequences and assuming that *N*_e_ = 10^6^ for *D. melanogaster*. Second, we estimated the mutation rate per year based on the number of fixed differences between *D. melanogaster* and *D. simulans*, assuming a divergence time of 3 Mya [53]. The two mutation rates yield qualitatively similar limits for the age of the sweep. Using the polymorphism-based estimate of *u*, the 95% upper confidence limit for the age of the sweep is 1,875 years. Using the divergence-based estimate of *u*, the 95% upper confidence limit for the age of the sweep is 3,360 years. Both estimates suggest that the major *SD-Mal* haplotype expanded across Africa very recently, within the last few thousand years.

### Accumulation of Linked Lethal Mutations

We performed complementation tests among all pairwise combinations of the 12 *SD* chromosomes, producing 12 *SD*_i_/*SD*_i_ and 66 *SD*_i_/*SD*_j_ genotypes. Both minor *SD* chromosomes (*SD*-*BN19* and *SD*-*MD31*) are homozygous viable, but all ten *SD*-*Mal* chromosomes are homozygous lethal (Table 6). Crosses among *SD*-*Mal* chromosomes, however, show that all ten fall into unique complementation groups—none of the lethal mutations is shared among major *SD*-*Mal* chromosomes (Table 6). This distribution of lethal mutations supports a star-shaped genealogy: all of the lethal mutations must have arisen on the *external* branches of the genealogical history of the *SD*-*Mal* chromosomes in our sample. These complementation data also reveal that lethal mutations are significantly over-represented on *SD-Mal* chromosomes relative to wildtype chromosomes: 29% of wildtype second chromosomes are lethal or semi-lethal [54] versus 100% of *SD-Mal* chromosomes (Fisher's exact *P* = 0.0015). The large *In(2R)Mal* rearrangement on *SD-Mal* chromosomes provides a large non-recombining target for lethal mutations that can persist by hitchhiking with the *SD* system.

**Table 6:** Complemenation tests for all *SD*_i_/*SD*_j_ combinations.

	*SD-GN09*	*SD-KM87*	*SD-KM92*	*SD-KN20*	*SD-KY38*	*SD-KY91*	*SD-MD21*	*SD-NK04*	*SD-ZK178*	*SD-ZK216*	*SD-BN19*	*SD-MD31*
*SD-GN09*	**Lethal**	MF	MF	MF	MF	MF	MF	MF	MF	MF	MF	MF
*SD-KM87*	FF	**Lethal**	MF	**MS**	MF	MF	MF	MF	MF	MF	MF	MF
*SD-KM92*	FF	FF	**Lethal**	MF	MF	MF	MF	MF	**MS**	MF	MF	MF
*SD-KN20*	FF	FF	FF	**Lethal**	**MS**	MF	**MS**	**MS**	MF	MF	MF	MF
*SD-KY38*	FF	FF	FF	FF	**Lethal**	MF	MF	MF	**MS**	MF	MF	MF
*SD-KY91*	FF	FF	FF	FF	FF	**Lethal**	MF	MF	**MS**	MF	MF	MF
*SD-MD21*	FF	FF	FF	FF	FF	FF	**Lethal**	**MS**	**MS**	MF	MF	MF
*SD-NK04*	FF	FF	FF	FF	FF	FF	FF	**Lethal**	MF	MF	MF	MF
*SD-ZK178*	FF	FF	FF	FF	FF	FF	FF	FF	**Lethal**	**MS**	MF	MF
*SD-ZK216*	FF	FF	FF	FF	FF	FF	FF	FF	FF	**Lethal**	MF	MF
*SD-BN19*	FF	FF	FF	FF	FF	FF	FF	FF	FF	FF	Viable, MF, FF	MF
*SD-MD31*	FF	FF	FF	FF	FF	FF	FF	FF	FF	FF	FF	Viable, MF, FF

Above the diagonal, MS = male sterile and MF = male fertile; below the diagonal FF = female fertile.

### Fertility in *SD*_i_/*SD*_j_ Flies

For the 66 viable *SD*_i_/*SD*_j_ and 2 viable *SD*_i_/*SD*_i_ genotypes, we tested the fertility of both sexes. None of the 68 genotypes were female-sterile, but 10 were male-sterile (Table 6). *SD-ZK178* is male-sterile in combination with five other *SD* chromosomes; *SD-KN20* is male sterile in combination with four others; and *SD-NK04* is male-sterile in combination with *SD-ZK216*. The patterns of complementation for male fertility are complex. For instance, *SD-KY38* and *SD-MD21* complement one another and yet both fail to complement *SD-ZK178*. Similarly, *SD-ZK178* and *SD-NK04* complement one another and yet both fail to complement *SD-ZK216*. Assuming that male sterility results from male-sterile mutations on chromosome 2, the data in Table 6 require a circular complementation map with at least 10 unique lesions. A more plausible hypothesis, however, is that male sterility results not from linked male-sterile mutations but from interactions among different alleles at *SD* complex loci [32],[55]. Indeed, previous work has shown that deletion of one copy of *Sd* rescues sterility in otherwise male-sterile *SD*_i_/*SD*_j_ combinations, supporting a connection between distortion and sterility [55]. The complex patterns of fertility complementation in *SD*_i_/*SD*_j_ males cannot, however, be explained by intragenic complementation at the *Sd* locus, as the *Sd-RanGAP* sequences among *SD-Mal* chromosomes are identical, suggesting that interactions involving other *SD* loci must be involved.

## Discussion

Two major findings emerge from our analysis of the *SD* system. First, *SD* occurs in ancestral, African populations of *D. melanogaster* at a frequency similar to that of other populations worldwide. This discovery raises doubts about the Mediterranean-origins hypothesis. Second, the evolution and rapid spread of a newer, stronger *SD* chromosome has left a dramatic population genetic signature: a remarkably long haplotype, spanning more than 39% of chromosome 2—roughly 30 cM—that is both free of polymorphisms (Table 3, Figure 3) and differentiated from other chromosomes in the population (Table 4). These findings suggest that a new *SD* chromosome type endemic to Africa, *SD-Mal*, has swept across the continent sometime within the last few thousand years.

### *SD* in Africa

The Mediterranean-origins hypothesis is based on the geographic distribution of inversions on *SD* chromosomes: inversion-bearing *SD* chromosomes occur throughout the world; but both inversion-bearing and inversion-free, presumably ancestral, *SD* chromosomes occur in Spain and Italy [3],[4]. The presence of ancestral *SD* chromosomes suggests that the complex may have arisen in Spain or Italy or nearby. Our discovery of *SD* chromosomes in African populations of *D. melanogaster* raises questions about the Mediterranean-origins hypothesis. Did *SD* originate in the Mediterranean and subsequently invade sub-Saharan Africa via back-migration? Or did *SD* originate in Africa and then make its way to Europe (and the rest of the world) as part of the *D. melanogaster* out-of-Africa event, ∼15,000 years ago [25]–[28]? The presence of inversion-free *SD* chromosomes in Benin and Cameroon (*SD-BN19* and *SD-MD31*, respectively) would seem to make a sub-Saharan African origin as likely as a Mediterranean one. In either case, the fact that inversion-free *SD* chromosomes occur in both Africa and the Mediterranean suggests that *Sd-RanGAP* dispersed from one location to the other shortly after it originated and then subsequently acquired different inversions on different continents.

The relative youth of the *Sd-RanGAP* duplication makes distinguishing between sub-Saharan African and Mediterranean origins with the present data difficult. We cannot, for instance, precisely date the origin of *Sd-RanGAP* from *RanGAP* based on the five fixed differences (1 indel, 4 nucleotide changes) by assuming a simple neutral molecular clock for two reasons. First, we cannot exclude the rapid, non-neutral fixation of changes in *Sd-RanGAP*. Second, some (or all) of the five fixed differences may have been segregating as the ancestral *RanGAP* sequence that ultimately gave rise to *Sd-RanGAP*. This putative ancestral *RanGAP* haplotype may be missing from our population sample by chance, or because it was lost from the population, or because it does not occur in African populations. Determining the time and place of origin for the *SD* system will therefore require deeper resequencing of *Sd-RanGAP* and *RanGAP* from both Europe and Africa.

### Evolutionary Turnover of *SD* Chromosomes

The population genetic analyses revealed six striking patterns among *SD* chromosomes (Table 3; Figure 3): significant excesses of rare variants; significant excesses of high frequency derived variants; an unusual distribution of haplotype frequencies (10+2 or 10+1+1; Figure 3); exceedingly long-range LD; a complete absence of polymorphism in >8.1 kb spanning >39% of the length of *SD-Mal* chromosomes; and significant population genetic differentiation between *SD-Mal* and other chromosomes (Table 4). Together these observations suggest that *SD-Mal* has spread to high frequency among *SD* chromosomes in Africa sometime within the last 3,000 years. Why might one type of *SD* chromosome rise in frequency so quickly, apparently displacing other *SD* chromosomes? The answer seems straightforward: *SD-Mal* chromosomes distort more than *SD-BN19* and *SD-MD31* and recombine less over the length of 2R, perhaps preserving a favorable distortion-enhancing combination of alleles in the *In(2R)Mal* region. Similar displacement of one *SD* type (*SD-5*) by another (*SD-72*) appears to have occurred during a 30-year period in populations in Wisconsin [31]. Thus, the apparently stable equilibrium frequency of *SD* chromosomes in *D. melanogaster* populations worldwide (1–5%) appears to mask a dynamic turnover among competing *SD* chromosome types.

There are at least two, non-exclusive explanations for the turnover of *SD* chromosomes. First, the *SD* system may be sufficiently new that it has not yet reached a stable evolutionary equilibrium: older *Sd-RanGAP* bearing chromosomes are still being displaced by new ones, like *SD-Mal* in Africa or *SD-72* in North America [31], as predicted by theory [15]. Second, an ultimately stable evolutionary equilibrium for *SD* chromosomes may not exist: *SD* may be engaged in a perpetual coevolutionary conflict with the rest of the genome [17]. Indeed, there is considerable variation among populations in the frequency of insensitive *Rsp* ^i^ alleles [30],[31] and other unlinked genetic variants that affect distortion (*e.g.*, [24],[34]). Under this scenario, the rise of *SD-Mal* and decline of *SD-BN19* and *SD-MD31* could reflect a transitional phase in the genetic conflict in Africa: *SD-BN19* and *SD-MD31* may no longer cause distortion because they have come under the effective control of unlinked suppressors in the genome, whereas adaptive changes specific to *SD-Mal* chromosomes allow them to escape suppression.

The discovery of two *Sd-RanGAP* bearing chromosomes that fail to cause distortion is surprising—indeed, classical phenotypic screens for segregation distortion undoubtedly would have misclassified *SD-BN19* and *SD-MD31* as wildtype chromosomes. While these chromosomes may now be suppressed, there are four other possibilities. One is that *SD-BN19* and *SD-MD31* have experienced mutations causing a loss of distortion. Mutational disruption of the *Sd-RanGAP* sequence seems unlikely, however, as all five differences that distinguish *SD-BN19* and *SD-MD31* from *SD-Mal* are silent. A second possibility is that recombination has stripped *SD-BN19* and *SD-MD31* chromosomes of essential modifiers required for distortion. Wildtype chromosomes that carry *Sd-RanGAP* transgenes but lack upward modifiers cause either very weak or even no distortion [37]. However, both of these scenarios—disruption by mutation or recombination—require that we explain the seemingly improbable coincidental loss of distortion by two identical, and relatively rare, *Sd-RanGAP* haplotypes. A third possibility is that *SD-BN19* and *SD-MD31* are not “*SD* chromosomes” but rather ancestral *Sd-RanGAP*-bearing chromosomes that never caused drive. This scenario would imply that *SD* chromosomes evolved from a neutral, non-driving ancestral haplotype: *Sd-RanGAP* arose as new duplication, drifted to sufficiently high frequency to become established via migration in Europe and in Africa, and then subsequently recruited genetic modifiers that conferred distortion. This history, if true, implies that African and non-African *SD* chromosomes independently acquired convergent distorting gene complexes. A final possibility is that *SD-BN19* and *SD-MD31* may cause distortion but not in the particular genetic backgrounds used in our assay. Further genetic analyses are required to distinguish these possibilities.

### Epistatic Selection Shapes Variation on *SD-Mal* Chromosomes

The long *SD-Mal* haplotype spans *Sd-RanGAP*, region 43E (locus *J*), and the *In(2R)Mal* inversions (*K*, *E*, *F*, and *G*; Figure 3) but does not extend distal to *Sd-RanGAP* on 2L or distal to *In(2R)Mal* on 2R. The structure of the *SD-Mal* haplotype probably reflects the hitchhiking effects of epistatic selection. First, consistent with the lack of loci known to affect distortion distal to *Sd-RanGAP*, *SD* and *SD* ^+^ chromosomes are free to recombine without consequence on the distal part of 2L, preventing LD there [4],[29]. Second, although *In(2R)Mal* suppresses recombination within the inverted regions, there is opportunity for crossing over in the interval between the *SD* complex loci (*Sd*, *E(SD)*, and *Rsp*) and the proximal breakpoint of the *In(2R)Mal*. The perfect LD across this interval suggests that strong epistatic selection maintains the association between the *SD* loci and the *In(2R)Mal* inversions. In principle, double-recombinants in the interval between centromeric *SD* loci and *In(2R)Mal* could preserve their association, but these may be rare events relative to the strength of epistatic selection favoring *SD-Mal*. Thus, positive epistatic selection on the *SD-In(2R)Mal* genotype may have caused hitchhiking effects to dominate the intervening sequence between them, explaining the skewed frequency spectrum, LD and lack of variability on *SD-Mal* chromosomes in region 43E (locus *J*). It is also possible that epistatic selection directly preserves an association with a *M(SD)* allele in the *SD-In(2R)Mal* interval [20], but we do not yet know if *SD-Mal* carries *M(SD)*. Third, inversions on 2R have been interpreted as tightening the association between *SD* and *St(SD)*, a modifier (or region of polygenic modifiers; ref. [56]) that increases the strength of distortion, putatively located near the tip of 2R [21],[22]. The fact that we fail to detect LD between *SD* and loci in cytological regions 58–59 (*H* and *I*; Figure 3) suggests that either no *St(SD)* loci reside in (or distal to) regions 58–59 as previously reported [22] or that no such *St(SD)* loci enhance distortion on *SD-Mal* chromosomes. It is important to note that *St(SD)*, like *M(SD)*, was characterized from non-African *SD* chromosomes; African *SD* chromosomes may carry a distinct set of linked modifiers.

### Explaining the Global *SD* Equilibrium

Although there appears to be competition among *SD* chromosomes, the overall frequency of *SD* in populations throughout the world is remarkably similar (1–5%; but see ref. [24]). Considering that different populations have experienced different environments, genetic backgrounds, and demographic histories, the seemingly stable frequency of *SD* suggests that its equilibrium is the result of strong deterministic forces. What prevents *SD* from reaching higher frequencies or even fixation? Three factors limit the spread of *SD*. First, as *SD* frequency increases, so does selection for insensitive *Rsp* ^i^ alleles and other genetic suppressors. Second, as *SD* frequency increases, intrinsically male-sterile *SD*_i_/*SD*_j_ genotypes become more common, placing an upper-limit on the spread of *SD* (Table 6; ref. [32]). Third, *SD*/*SD* ^+^ males have been shown to suffer reduced male fertility, as might be expected when 50% of sperm are destroyed [9]. Finally, many *SD* chromosomes worldwide, including the new *SD-Mal* chromosomes, carry linked recessive lethal and other deleterious mutations (Table 6). The large non-recombining, inverted blocks of chromosome that become associated with *SD* present a large mutational target. Without recombination, linked recessive lethal and other deleterious mutations are able to persist by hitchhiking with *SD*. It remains unclear if these factors are sufficient to explain the distortion-selection balance that causes the frequency of *SD* to settle at 1–5% in *D. melanogaster* populations worldwide.

### Conclusions

The hitchhiking effects of selfish meiotic drive gene complexes have shaped patterns of DNA sequence variability in at least five other cases: four selfish X chromosome systems (one in *Drosophila pseudoobscura* [57], two in *Drosophila simulans* [58],[59], and one in *Drosophila recens* [60]) that drive in the male germline and a selfish autosomal centromere that drives in the female germline of the monkeyflower, *Mimulus guttatus* [61]. Like *SD*, all five of these drive systems are associated with haplotypes of reduced variability and three show long-range LD—the signatures of partial selective sweeps. Notably, all five are balanced polymorphisms in which the drive elements are prevented from going to fixation by modifiers or countervailing selection. It is important to note that these well characterized drive systems may not be representative, as there is a clear detection bias: to be discovered and characterized, drive systems must be conspicuous (*e.g.*, causing strong drive or distorting sex ratios) and segregate within populations (*i.e.*, balanced) [7]. But what about those drive elements that are not balanced and thus able to spread to fixation? These would also invade when concentrated in the centromeric regions of autosomes or on sex chromosomes (little or no crossing over occurs between the X and Y) and then sweep through populations, causing *complete* rather than partial selective sweeps. The extent to which hitchhiking effects of selfish meiotic drive systems contribute to overall patterns of DNA sequence variation, reducing variability around centromeres and on sex chromosomes (*e.g.*, ref. [62]), remains to be determined.

## Methods

### PCR-Screen for *SD* Chromosomes

We used a molecular assay to screen for *SD* chromosomes in a collection of 452 isofemale lines from across sub-Saharan Africa, kindly provided by Drs. John Pool, Charles Aquadro and Andy Clark (Cornell University). We used a single-reaction PCR assay involving three primers, a forward primer (F) and two reverse primers (R1 and R2): F = TTTGGAGACTGCCTGATCAAAACTAATG; R1 = CAACGTCGCGGAGGAGACTGCCTATGT; R2 = CGTGTTCTGAGCGTTTCGCACAGTGTAT. One primer pair (F-R1) amplifies a 463-bp fragment from the parent gene, *RanGAP* (a positive control), and the other (F-R2) amplifies a 353-bp *SD*-specific fragment that spans the breakpoint of the *Sd-RanGAP*-*RanGAP* junction (Figure 1B). Only one amplicon results from flies that lack *SD* chromosomes and two result from flies that carry *SD* (Figure 1B).

### Extracting *SD* Chromosomes

Isofemale lines found to be *SD*-positive by PCR assay could be homozygous *SD*/*SD* or heterozygous *SD*/*SD^+^*. We therefore extracted *SD* chromosomes onto a common genetic background, then maintained homozygous viable *SD* chromosomes as homozygous stocks, and maintained homozygous lethal *SD* chromosomes over the *CyO* balancer chromosome. To extract *SD* chromosomes, we crossed 3–5 *w* ^118^; *In(2LR)Gla*, *wg* ^Gla-1^ *Bc* ^1^/*CyO* (hereafter, *w* ^118^; *Gla*/*CyO*) virgin females to 3–5 males from the *SD*-positive isofemale lines. We then collected 5 white-eyed *CyO* sons and individually backcrossed them to 5–10 *w* ^118^; *Gla*/*CyO* females. Once larvae appeared in the backcross vials, we PCR-tested the 5 white-eyed *CyO* sons for *SD* (see above) and retained progeny from a single *SD*-positive male. We then crossed *w* ^118^/*w* ^118^; *SD*/*CyO* virgin daughters to *w* ^118^; *SD*/*CyO* sons. If the *SD* chromosome was homozygous viable, we used the progeny to establish a *w* ^118^; *SD*/*SD* stock; if the *SD* chromosome was homozygous lethal, we maintained a *w* ^118^; *SD*/*CyO* stock. Last, we confirmed that all of the final stocks carried the *SD* chromosome by PCR assay.

### Inversion-Typing *SD* Chromosomes

Many *SD* chromosomes possess one or more inversions on chromosome 2 (reviewed in ref. [2]). To determine the inversion types of *SD* chromosomes, we examined polytene chromosomes from larval salivary gland squashes. We crossed virgin *cn bw* females to *SD* males to generate larvae; *cn bw* chromosomes have standard arrangement second chromosomes. Salivary glands were dissected from F_1_ larvae in 1% Na-citrate hypotonic solution on siliconized slides and then transferred and fixed for 10–15 seconds in 45% acetic acid. The dissections were stained with 1% lacto-aceto-orcein for 25–35 minutes. We determined inversion breakpoints by comparing photographs with the standard maps of chromosome 2.

### Complementation Tests among *SD* Chromosomes

We performed complementation tests between all pairwise combinations of *SD* chromosomes. For all homozygous lethal *SD* chromosomes, we tested the viability of all *SD*_i_/*SD*_j_ combinations by crossing five *SD*_i_/*CyO* virgin females to 3–5 *SD*_j_/*CyO* males. If *CyO* ^+^ progeny appear, then the lethality of *SD*_i_ and *SD*_j_ chromosomes must map to different complementation groups. We also tested the male and female fertility of viable *SD*_i_/*SD*_j_ combinations. At least two replicates each of 3–5 *SD*_i_/*SD*_j_ males and 3–5 virgin *SD*_i_/*SD*_j_ females were crossed to OreR virgin females and males, respectively. *SD*_i_/*SD*_j_ flies that produced larvae were considered fertile, whereas those that failed to produce any progeny over multiple replicates were considered sterile.

### Estimating the Strength of Segregation Distortion

We estimated the strength of distortion for each *SD* chromosome by measuring the rate of transmission, *k*, of the *SD* chromosome through heterozygous *SD*/*Gla* males. In preliminary work, we screened a series of balancer chromosomes (*Bal*) for sensitivity to distortion by assaying transmission from *SD-5*/*Bal* males. *SD-5* is a well-characterized, non-African *SD* chromosome. These crosses revealed that the *In(2LR)Gla* chromosome (hereafter, *Gla*) carries a sensitive *Rsp* ^s^ allele. *Gla* is an effective balancer of most of the second chromosome and carries a dominant eye-phenotype marker. We estimated *k* by individually crossing five *SD*/*Gla* males of each *SD* chromosome to five 3–5 day old *cn bw* virgin females each. After four days, each cross was transferred to a fresh food vial every fourth day. We then scored all progeny emerging until 20 days after the parents were removed from each of the four vials.

The rate of transmission of *SD* to progeny depends both on the strength of distortion and on the relative viability of the *SD* chromosome. Therefore, to distinguish the strength of distortion from relative viability, we measured the rate of transmission of *SD* chromosomes through heterozygous *SD*/*Gla* females. As distortion is male-specific, the rate of transmission of *SD* through females allows estimation of *SD* relative viability. By using the *Gla* balancer to minimize recombination on the second chromosome in females, we could estimate the viability of intact *SD* chromosomes like those transmitted through males (which lack recombination in *D. melanogaster*). For each *SD* chromosome we set up three replicate crosses of five 3–5 day old *SD*/*Gla* virgin females with three 3–5 day old *cn bw* males. After four days, each cross was transferred to fresh vial every fourth day. We used our estimates of relatively viability to estimate a corrected strength of distortion, k*, following ref. [63].

### Sequencing of *Sd-RanGAP*

To sequence the new *Sd-RanGAP* duplicate gene, we first isolated *SD* chromosomes in heterozygous state over a chromosomal deficiency, *Df(2L)Sd77*, which deletes the 37D1–37D2;38C1–38C2 region including the *RanGAP* locus. After isolating genomic DNA from *SD*/*Df(2L)Sd77* flies, we PCR amplified two fragments from the *Sd-RanGAP* region with two sets of primers. All PCR products therefore come from the *SD* chromosome. The first set amplifies a 2,994-bp fragment from the 5′-half of *Sd-RanGAP*. The forward primer (F4) binds the distal intergenic region between *Sd-RanGAP* and the neighboring gene *CG10237*; the reverse primer (R4) binds in intron 1 of *Sd-RanGAP* (which, on the reverse strand, is exon 2 of *Hs2st*). The second primer set amplifies a 2,410-bp fragment from the 3′-half of *Sd-RanGAP* with a 280-bp overlap with the first fragment. The forward primer (F6) binds in the first intron of *Sd-RanGAP* (which, on the reverse strand, is intron 2 of *Hs2st*); the reverse primer (R6) binds the intergenic region between *Sd-RanGAP* and *RanGAP*. Both the R4 and F6 primers bind two genomic locations in flies with *SD* chromosomes. First, R4 binds the first intron of *Sd-RanGAP* and the homologous sequence of the parent gene *RanGAP*. However, when the F4-R4 primer pair is used and PCR extension times are constrained, only product from the first R4 binding location results. Second, F6 binds the first intron of *Sd-RanGAP* and the homologous sequence of *RanGAP*. However, when the F6-R6 primer pair is used, only the 3′-half of *Sd-RanGAP* is amplified. We used Exo-SAP to clean PCR products and then sequenced both strands of the PCR products using internal sequencing primers (Table S1), BigDye Terminator chemistry, and standard cycle sequencing protocols. All sequences were manually edited using *Sequencher* v. 4.5 (Gene Codes). We obtained outgroup sequences via BLAST searches of the *D. simulans* genome [62].

### Sequencing Non-Coding Regions on Chromosome 2

In addition to *Sd-RanGAP*, we sequenced the parent gene and eight non-coding regions across chromosome 2 from a collection of *SD* chromosomes and from 10 wildtype chromosomes from Zimbabwe. As many *SD* chromosomes, and some wildtype ones, are homozygous lethal (see RESULTS), we could not make homozygous lines for sequencing for all stocks. Instead, for homozygous lethal lines, we used deficiencies to produce flies hemizygous for the focal chromosomal regions. The eight regions ranged in size from 567–874 bp long (Table 3). We sequenced fragments from the proximal intergenic region of *tup* (cytological position = 37B; deficiency used for hemizygous flies = *Df(2L)Exel7073*); a large intron from *CG30947* (43E; *Df(2R)Exel6054*); the distal intergenic region of *Myd88* (45C; *Df(2R)Np3*); the proximal intergenic region of *off-track* (48D6; *Df(2R)BSC39*); the proximal intergenic region of *scab* (51E; *Df(2R)Jp1*); the proximal intergenic region of *staufen* (55B; *Df(2R)Pcl7B*); a large intron of *plexus* (58E4–8; *Df(2R)Exel7173*); a large intron of *CG34372* (59E1; *Df(2R)bw-S46*). To sequence the parent gene, *RanGAP*, from the 10 wildtype chromosomes, we used the *Df(2L)Sd77*.

### Population Genetic Analyses

We performed most population genetic analyses using *DnaSP* [64]. Probability values for Tajima's *D* and Fay and Wu's *H* were obtained from 10,000 coalescent simulations with no recombination, conditioning on the observed *θ*. For coalescent-based haplotype configuration tests we used the *haploconfig* software [48].

## Supporting Information

Table S1 Primer sequences for sequencing *Sd-RanGAP*.
(0.06 MB DOC)
