# Supplementary material for: Large-Scale Selective Sweep among Segregation Distorter Chromosomes in African Populations of Drosophila melanogaster
Source: PLoS Genet. 2009 May 1;5(5):e1000463. doi: 10.1371/journal.pgen.1000463 (PMC2668186; doi:10.1371/journal.pgen.1000463)
Supplement: Table S1 — Primer sequences for sequencing Sd-RanGAP. (0.06 MB DOC) [file pgen.1000463.s001.doc]

**Supplementary Materials for:**

Presgraves, D.C., P. Gerard, A. Cherukuri and T.W. Lyttle, “Large-scale selective sweep among *Segregation Distorter* chromosomes in African populations of *Drosophila melanogaster*“.

**Primer sequences for sequencing *Sd-RanGAP***

For DNA sequencing, *Sd-RanGAP* was amplified as two overlapping fragments from genomic DNA extracted from either homozygous *SD/SD* or hemizygous *SD*/*Df(2L)Sd77* flies (see Methods).

|  |  |  |
| --- | --- | --- |
|  |  |  |
| Primer | Forward / Reverse | *Sd-RanGAP* primer sequences |
|  |  |  |
|  |  |  |
| *Fragment 1* |  |  |
| *F4 | F | ATGCACTGCGTGCATTGCACATATGTATTGTACG |
| 1.18 | F | ATGGTTCACTGAGCTCAGCAT |
| 1.14 | F | ATTCTCATCTGCCACTTGCG |
| 1.2 | R | CGAATGGCTAAATGGCGGAATGGCG |
| 2.1 | F | TGCGCTTGCCTGAGCCCATTCTGCG |
| 2.2 | R | CGTACACTTAAGCGCTACCTAAGACTTTAG |
| 3.1 | F | CAGACAATTGTTGCTAGATATTGCTTACGATG |
| 3.2 | R | CAGATGTACGAGTTTGTGGATCTGCTCGAG |
| 4.1 | F | AGCTTGGAAGATGTCACCCGCAGATGAG |
| 4.2 | R | ACCGACCGAATTTAGTCCGCAAGAAGG |
| 5.1 | F | AGGTTAATACCTTGATGCTCTAC |
| *R4 | R | ACTTCGAGGAGCACCTAGTGGTGCTTTACAATCG |
|  |  |  |
|  |  |  |
| *Fragment 2* |  |  |
| *F6 | F | AGACGGGCTCTGCTGGATTGGCTAGATGTTACCTACTTTG |
| 6.2 | R | CGCTGATATGAGCTACCGTGCTGACCT |
| 7.1 | F | AGGTCAGCACGGTAGCTCATATCAGCG |
| 7.2 | R | CACCCAAACTTACCATCGGCAGCTGT |
| 8.1 | F | ACAGCTGCCGATGGTAAGTTTGGGTG |
| 8.2 | R | CGGCAAGGGCCTCGACGCCTTCGATGT |
| 9.1 | F | ACATCGAAGGCGTCGAGGCCCTTGCCG |
| *R6 | R | ATCTTTAGAGCTCTTAACTCGAATCGGTCGCTTATAATG |
|  |  |  |
|  |  |  |
| * = PCR primers | |  |
